# Neurological Complications of Coronavirus Disease (COVID-19): Encephalopathy

**DOI:** 10.7759/cureus.7352

**Published:** 2020-03-21

**Authors:** Asia Filatov, Pamraj Sharma, Fawzi Hindi, Patricio S Espinosa

**Affiliations:** 1 Neurology, Charles E. Schmidt College of Medicine, Florida Atlantic University, Boca Raton, USA; 2 Neurology, Marcus Neuroscience Institute, Boca Raton Regional Hospital, Boca Raton, USA

**Keywords:** covid-19, encephalopathy

## Abstract

Coronavirus disease 2019 (COVID-19) is a pandemic. Neurological complications of COVID-19 have not been reported. Encephalopathy has not been described as a presenting symptom or complication of COVID-19. We report a case of a 74-year-old patient who traveled from Europe to the United States and presented with encephalopathy and COVID-19.

## Introduction

Coronavirus disease (COVID-19) was first detected in December 2019 in China and has rapidly spread to the rest of world. The World Health Organization (WHO) has recently declared COVID-19 a pandemic, with more than 180,000 reported cases to date. COVID-19 is a novel corona virus that probably emerged from an animal source, which is now spreading rapidly from person to person. The typical symptoms of COVID-19 can range from mild to severe respiratory illness. The most common symptoms that have been reported thus far are fever, cough, and shortness of breath. The elderly population, especially those with underlying medical problems like chronic bronchitis, emphysema, heart failure, or diabetes, are more likely to develop serious illness [1,2].

Neurological complications in COVID-19 infected patients have not been widely reported. Since elderly patients with chronic medical conditions are at an increased risk of altered mental status in the setting of acute infections, patients with COVID-19 infection can also present with acute encephalopathy and changes in their level of consciousness. Here we report a case of a patient who presented with encephalopathy and was found to be infected with COVID-19.

## Case presentation

**Figure 1:**
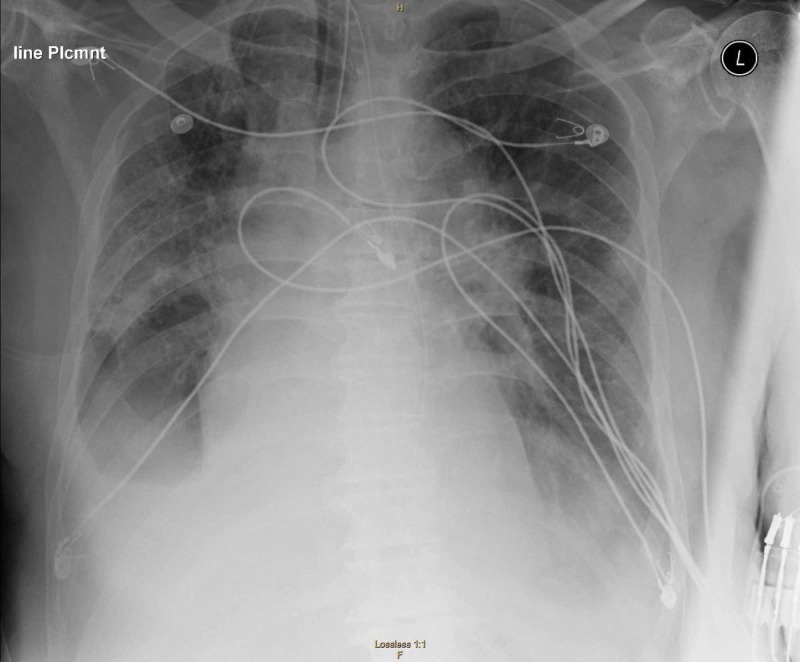
Chest X-ray shows multifocal airspace opacities and “ground-glass opacities”, characteristic signs of COVID-19 infection.

A 74-year-old male with past medical history of atrial fibrillation, cardioembolic stroke, parkinson disease, chronic obstructive pulmonary disease (COPD), and recent cellulitis presented to the emergency department with a chief complaint of fever and cough. The patient had a full workup, including routine labs and chest x-ray, which were nonrevealing. The patient was discharged home under the suspicion that this was an exacerbation of his COPD. The patient went home on oral antibiotics. The patient returned to the emergency room (ER) within 24 hours with worsening symptoms, including headache, altered mental status, fever, and cough. The patient is originally from the Netherlands and presented to our hospital seven days after arriving to the United States. The patient was admitted to the hospital for further workup. All protective measures and precautions for suspected COVID-19 infection were taken. The patient was placed in isolation. Repeat chest x-ray demonstrated small right pleural effusion with bilateral ground glass opacities (see Figure 1). CT chest revealed patchy bibasilar consolidations and subpleural opacities. Both throat sputum and nasopharyngeal cultures were negative for strep. Blood cultures were negative, and urine analysis was negative. Influenza A and B tests were negative.

**Figure 2:**
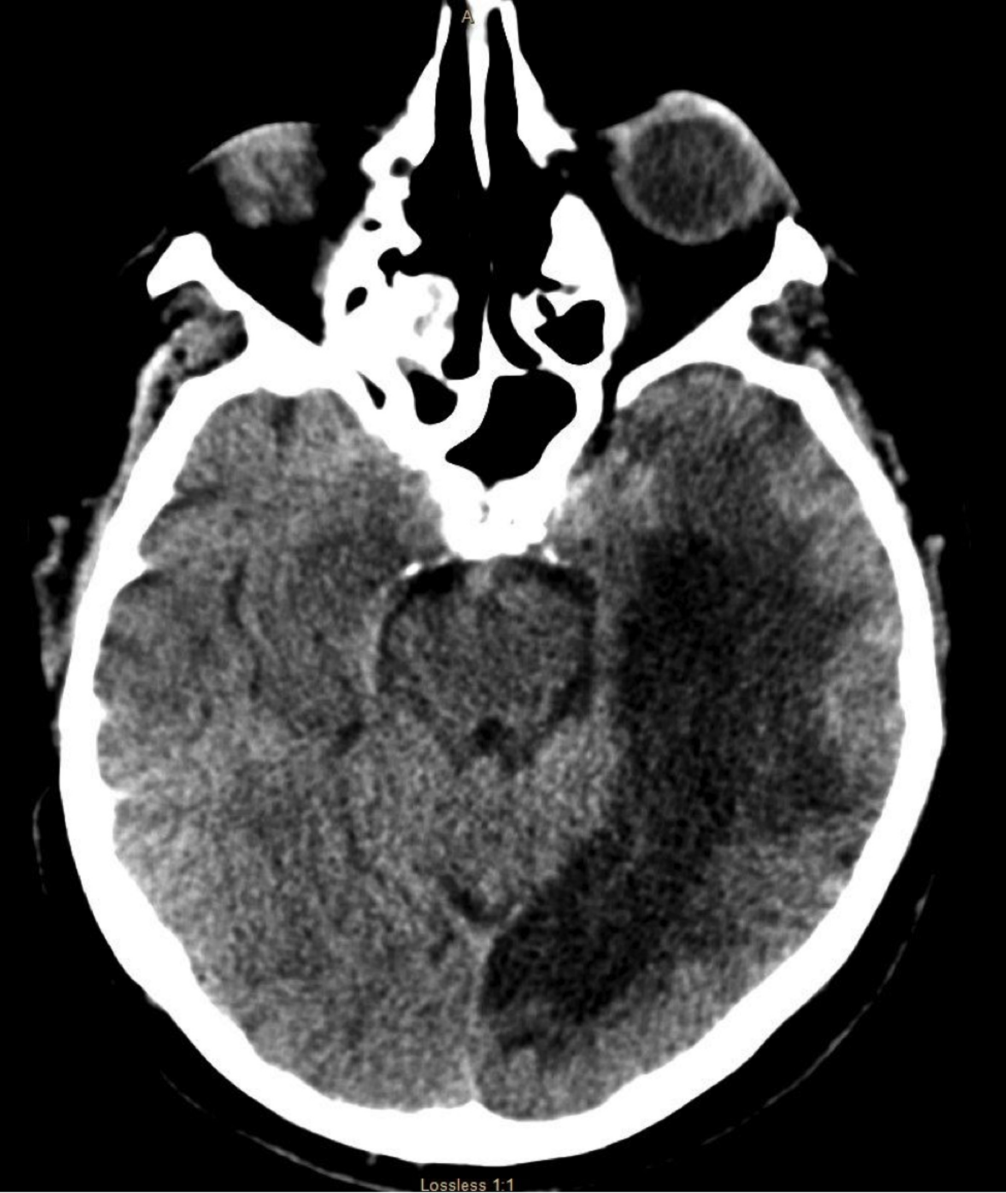
CT scan of the head that shows no acute changes. There is an area of hypodensity in the right temporal region, consistent with patient know prior history of left PCA embolic stroke

**Figure 3:**
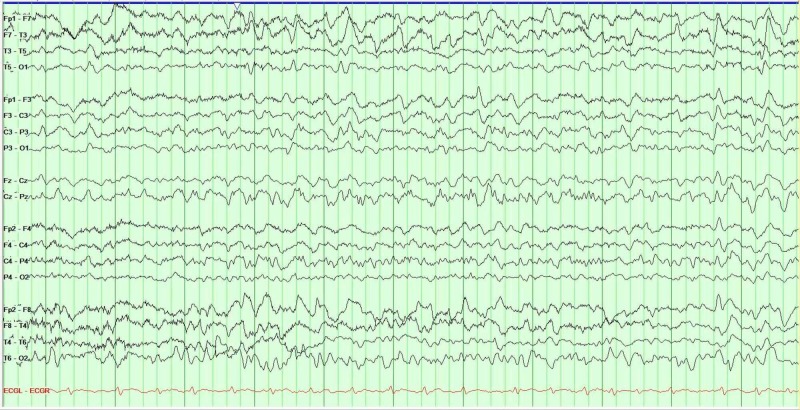
EEG shows diffuse slowing and focal slowing sharply contoured waves in the left temporal region. These findings are consistent with an encephalopathy, focal left temporal lobe dysfunction, and possible epileptogenicity.

Due to the severe alteration in mental status, neurology was consulted. Upon examination, the patient was found encephalopathic, nonverbal, and unable to follow any commands; however, he was able to move all his extremities and was reacting to noxious stimuli. No nuchal rigidity was noted. A CT scan of the head and EEG were ordered immediately. The CT scan of the head showed no acute abnormalities. There was the presence of an area of encephalomalacia in the left temporal region, consistent with the prior history of embolic stroke (see Figure 2). The EEG showed bilateral slowing and focal slowing in the left temporal region with sharply countered waves (see Figure 3).

The patient was started on antiepileptic medication prophylactically given the possibility of subclinical seizures in this patient with an area of encephalomalacia and epileptiform discharges in the right temporal region. The patient was treated empirically with vancomycin, meropenem, and acyclovir. A lumbar puncture did not reveal any evidence of central nervous system infection (see Table 1). Due to his progression in symptomatology, he was then tested for COVID-19 and found to be positive. The patient developed respiratory failure and required intubation and was transferred to the ICU. Based on anecdotal experience from other medical centers, the patient was started on hydroxychloroquine and lopinavir/ritonavir, and was continued on broad-spectrum antibiotics. The patient currently remains in the ICU, critically ill with poor prognosis. 

**Table 1:** CSF analysis: CSF studies show no evidence of CNS infection. CMV, cytomegalovirus; CNS, central nervous system; CSF, cerebrospinal fluid; HSV, herpes simplex virus; PCR, polymerase chain reaction

CSF studies	
Appearance	Clear
Color	None
White blood cells	4
Red blood cells	0
CSF glucose	75
CSF protein	68
Albumin	36
HSV PCR	Not detected
CMV PCR	Negative
Respiratory syncytial virus	Negative

## Discussion

COVID-19 is a pandemic than ranges from mild disease with nonspecific signs and symptoms to acute respiratory symptoms to severe pneumonia with respiratory failure and septic shock. Current evidence suggests that COVID-19 patients commonly had neurological symptoms manifested as acute stroke (6%), consciousness impairment (15%), and skeletal muscle injury (19%) [3]. Elderly patients with chronic conditions are at an increased risk of altered mental status in the setting of acute infections. Since COVID-19 affects more the elderly and those with preexisting conditions, patients with prior neurological conditions and acute respiratory symptoms are at an increased risk of encephalopathy on initial presentation. The cerebrospinal fluid studies in our patient were normal; therefore, COVID-19 does not cross the blood-brain barrier and does not cause meningitis or encephalitis. Our case highlights the importance of identifying encephalopathy as a presenting sign of COVID-19. Patients with COVID-19 testing positive with common features of cough, fever, and shortness of breath can present to the ER with encephalopathy, or can develop encephalopathy during their hospital stay. Neurologist will be consulted, and practitioners have to know that this may encounter in the acute setting. Given that there is limited data on neurological symptoms, health care providers benefit from accurate and real-life data to better treat their patients. If patients with neurological conditions are not considered to have COVID-19, this may present a nationwide issue to health care team members treating patients and in turn the general public if they are discharged and further exposed to other people.

## Conclusions

Health care providers should be aware that patients with COVID-19 can present with encephalopathy in the acute setting and during hospitalization. 
